# Deconstruction of a multi-strain *Bacillus*-based probiotic used for poultry: an in vitro assessment of its individual components against *C. perfringens*

**DOI:** 10.1186/s13104-023-06384-1

**Published:** 2023-06-22

**Authors:** Vinicius Buiatte, Maria Schultheis, Alberto Gino Lorenzoni

**Affiliations:** 1 Department of Animal Science, College of Agricultural Sciences, The Pennsylvania State University, University Park, PA USA; 2 College of Veterinary Medicine, The Ohio State University, Columbus, OH USA

**Keywords:** Probiotics, Poultry, Competitive exclusion, Co-culture

## Abstract

**Objective:**

Probiotics have been used in poultry production to improve the performance and health of chickens raised without antibiotics. The combination of different probiotic strains has been used with the hope of conferring multiple benefits to the host. However, the inclusion of several strains does not necessarily boost benefits. There is a lack of studies that compare the efficacy of multi-strain probiotics to their individual components. In this study, the effects of a *Bacillus*-based probiotic product mix containing *B. coagulans*, *B. licheniformis*, *B. pumilus*, and *B. subtilis* against *Clostridium perfringens* were tested in vitro using a co-culture method. The individual strains and different combinations of the strains used in the product were also tested against *C. perfringens*.

**Results:**

The probiotic product mix tested in this study did not show effects against *C. perfringens* (*P* = 0.499). When tested individually, the strain of *B. subtilis* was the most efficient strain to decrease *C. perfringens* concentrations (*P* ≤ 0.01), and the addition of other *Bacillus* species strains significantly decreased its efficacy against *C. perfringens*. We concluded that the probiotic mix of *Bacillus* strains used in this study (*B. coagulans*, *B. licheniformis*, *B. pumilus* and *B subtilis*) was not effective in decreasing *C. perfringens* concentrations in vitro. However, when deconstructing the probiotic, the strain of *B. subtilis* alone or combined with the strain of *B. licheniformis* were effective against *C. perfringens*. This suggests that the anticlostridial properties of the particular strains of *Bacillus* used in this study were negatively affected when combined with other *Bacillus* spp. strains.

## Introduction

Probiotics are microorganisms that impart benefits to the host. Probiotics as single species or as a combination of multiple species can be used as feed additives to improve health and performance in poultry [1, 2]. The mode of action of probiotics is not entirely understood [3]. Studies have shown that probiotics used in poultry could prevent the growth of pathogenic bacteria, improve the gastrointestinal structure, and modulate the immune system [4]. Probiotics can be used as an alternative to antibiotics for the control of necrotic enteritis, a disease caused by toxin-producing *C. perfringens* that decreases growth performance in poultry, leading to economic losses [5].

Species of *Bacillus*, such as, *B. subtilis*, *B. licheniformis*, *B. coagulans*, *B. clausii*, *B. pumilus*, and *B. cereus*, have been considered good probiotic candidates because they are spore-forming and can survive in high temperatures and at low pH. These features increase the survivability of beneficial bacteria during feed processing and storage, and during their passage through the gastrointestinal tract [6].

*Bacillus* spp. probiotics have shown antagonistic effects against selected bacteria in vitro and are effective alternatives to antibiotics for the control of necrotic enteritis in vivo [7, 8]. In particular, *B. subtilis* is probably the best-characterized species for the control of *C. perfringens* and has been shown to be an effective probiotic in in vitro and in vivo experiments [9, 10]. *B. subtilis* has been used individually or combined with other species, however, it is not clear whether the inclusion of other *Bacillus* species results in enhanced probiotic efficiency against pathogens. Studies that compare the efficacy of a particular probiotic strain used as single-strain versus a multi-strain product are often missing [11].

The objectives of this study were to evaluate the inhibitory effect of a proprietary probiotic mix containing four strains of *Bacillus* spp. against *C. perfringens in vitro*, and to test all its individual strains and some of their possible combinations against *C. perfringens in vitro.*

## Materials and methods

### Experimental design

A proprietary probiotic product developed to improve poultry performance was tested for its ability to inhibit *Clostridium perfringens in vitro* following a modified method previously published [12]. The anti-clostridial effects of *Bacillus subtilis* (BS), *Bacillus licheniformis* (BL), *Bacillus coagulans* (BC) and *Bacillus pumilus* (BP) were assessed individually or in the combinations shown in Table 1, using a co-culture method.

A total of three experiments were performed in triplicates. For each experiment, a control treatment, consisting of a pure culture of *C. perfringens* was included to compare bacterial counts between the probiotic treatment and the control.

**Table 1:** Experiments performed with different combinations of *Bacillus* spp. against *Clostridium perfringens* ATCC 13,124™ using an in vitro co-culture method

Experiment	Co-culture
1	Commercial product containing *B. subtilis*, *B. pumilus*, *B. coagulans*, and *B. licheniformis* + *C. perfringens*
2	*B. subtilis + C. perfringens* *B. pumilus + C. perfringens* *B. licheniformis + C. perfringens* *B. coagulans + C. perfringens*
3	*B. subtilis + B. pumilus + C. perfringens* *B. subtilis + B. licheniformis + C. perfringens* *B. subtilis + B. coagulans + C. perfringens* *B. subtilis + C. perfringens*

### Preparation of C. perfringens inoculum

A primary culture of *C. perfringens* ATCC 13,124 ™ was started by inoculating a fresh colony into 10 mL of Tryptic Soy Broth (TSB, Neogen®, United States) followed by incubation for 24 h at 37 °C under anaerobic conditions. Anaerobiosis was achieved by adding a pouch of the AnaeroPack® System (Mitsubishi Gas Chemical America®, United States) into a hermetic chamber. The cultures of *C. perfringens* were standardized to reach a concentration of 1 × 10^8^ CFU/mL.

An inoculum of *C. perfringens* was created by diluting the primary culture one thousand-fold (1:1,000) to reach a target concentration of 1 × 10^5^ CFU/mL. Tenfold serial dilutions were completed and plated onto Shahidi-Ferguson Perfringens (SFP) agar (Millipore®, Germany) for the determination of bacterial counts.

### Preparation of Bacillus spp. inoculum

The product mix and the isolates of *Bacillus* spp. strains were provided by the manufacturer in a lyophilized form. The probiotics were suspended in sterile saline for their use in the co-culture model. Bacterial concentrations were determined by performing tenfold dilutions, plating onto Anaerobic Blood Agar (Remel™, United States), and incubating at 37 °C for 24 h under anaerobic conditions.

### Co-cultures

The co-cultures were created by adding 1 mL of the *C. perfringens* inoculum and 5 µL each *Bacillus* spp. inoculum into 3 mL of TSB. For the commercial product, 20 µL of the inoculum were used. The final target concentration of *Bacillus* in the co-culture was 2.5 × 10^5^ CFU/mL of each *Bacillus* strain to resemble the in-feed concentration of the probiotic recommended by the manufacturer. The final concentration of *C. perfringens* in the co-culture was 2.5 × 10^4^ CFU/mL to represent the normal range of *C. perfringens* in the gastrointestinal tract of healthy birds (10^2^-10^4^ CFU/g of digesta) [13]. Co-cultures were incubated at 37 °C for 24 h under anaerobic conditions. In every experiment, a control treatment was created using the same procedure, with the *Bacillus* spp. inoculum replaced with sterile saline.

After incubation, ten-fold serial dilutions of the co-cultures and the control treatment were used to determine the concentrations (CFU/mL) of *C. perfringens*. The dilutions were plated onto SFP agar plates.

### Statistical analysis

The one-way ANOVA was used to test the effects of the treatments on the concentration of *C. perfringens* in Minitab® 20 (Minitab Inc., United States). Treatment was assumed to be a fixed effect. Log base ten transformations were performed for the response variable (*C. perfringens* concentration) to stabilize the variances. Fisher’s least significant difference (LSD) was used to separate the means, and a difference in the mean of *C. perfringens* concentrations was claimed when *P* ≤ 0.05. Results are presented as mean ± standard error (SE).

## Results

In Experiment 1, the commercial probiotic product containing *B. coagulans*, *B. licheniformis*, *B. pumilus* and *B. subtilis* did not reduce the concentration of *C. perfringens* (7.35 log_10_ CFU/mL ± 0.165) compared to the control (7.07 log_10_ CFU/mL ± 0.02) (*P* = 0.499).

In Experiment 2, *B. coagulans*, *B. licheniformis*, *B. pumilus* and *B. subtilis* produced different effects against *C. perfringens* (*P <* 0.01) (Table 2). The most effective species against *C. perfringens* was *B. subtilis*, with a 6-Log_10_ reduction (10^2^) compared to the control (10^8^). Individually, *B. coagulans*, *B. pumilus*, and *B. licheniformis* produced a 1-Log_10_ reduction (10^7^) compared to the control treatment.

**Table 2:** The in vitro effect of single strains of *Bacillus* spp. on the concentration of *C. perfringens* using a co-culture method (Experiment 2)

Treatment	Average final *C. perfringens* concentration (Log_10_ CFU/mL) ± SE
BS + CP	2.81^d^ ± 0.07
BC + CP	7.71^c^ ± 0.02
BP + CP	7.86^b^ ± 0.01
BL + CP	7.93^b^ ± 0.02
Control (CP only)	8.69^a^ ± 0.03

*B. subtilis* (BS); *B. coagulans* (BC); *B. pumilus* (BP); *B. licheniformis* (BL); *C. perfringens* (CP).

CFU: colony-forming units.

Different superscripts in the same column denote statistical differences.

In Experiment 3, a significant difference was observed in the treatments containing *Bacillus subtilis* (*P* < 0.01) (Table 3). The treatments *B. subtilis* + *C. perfringens* (BS + CP) and *B. subtilis* + *B. licheniformis* + *C. perfringens* (BS + BL + CP) were the most effective against *C. perfringens* with a 5-Log_10_ reduction (10^2^) and a 3-Log_10_ reduction (10^4^), respectively, compared to the control (10^7^).

**Table 3:** The in vitro effect of different combinations of *Bacillus subtilis* with other *Bacillus* spp. on the concentration of *C. perfringens* using a co-culture method (Experiment 3)

Treatment	Average final *C. perfringens* concentration (Log_10_ CFU/mL) ± SE
BS + CP	2.69^b^ ± 0.0
BS + BL + CP	4.03^b^ ± 0.33
BS + BP + CP	6.02^a^ ± 1.17
BS + BC + CP	6.57^a^ ± 0.16
Control (CP only)	7.55^a^ ± 0.20

*B. subtilis* (BS); *B. coagulans* (BC); *B. pumilus* (BP); *B. licheniformis* (BL); *C. perfringens* (CP).

CFU: colony-forming units.

Different superscripts in the same column denote statistical differences.

## Discussion

Under the conditions used in this study, the tested multi-strain probiotic product containing BC, BS, BL and BP did not have significant anticlostridial effects in vitro. It is pertinent to underscore that this product was not designed to suppress the growth of *C. perfringens*. In this context, it is not surprising that the product did not offer anticlostridial activity. However, it is interesting that, some of the strains utilized in this probiotic mix were indeed effective against *C. perfringens* when individually tested. Notably, the strain of *B. subtilis* contained in the tested probiotic mix was the most effective species in decreasing *C. perfringens* concentrations *in vitro.* This result is in accordance with previous studies that tested the efficacy of *B. subtilis* strains against *C. perfringens in vitro* [8]. However, the efficacy of *B. subtilis* against *C. perfringens* was significantly reduced when *B. subtilis* was combined with the other *Bacillus* species, possibly because of antagonistic interactions between these strains.

Bacteriocins are peptides produced by bacteria that can inactivate other bacterial species, including species within the same genus [14]. *Bacillus* spp. are known to produce bacteriocins, such as mersacidin and sublancin [15], that are part of the lantibiotic family of peptides. This family of peptides can form pores in the bacterial cell membrane and inhibit cell wall formation, leading to cell death [16–18]. Although mechanisms of action were not investigated in the present study, we speculate that the addition of other *Bacillus* species strains in a co-culture with the *B. subtilis* strain may have suppressed the latter. Moreover, bacteria can modulate their development through quorum sensing systems when the availability of nutrients is scarce and bacterial populations are growing [19, 20]. It is also possible that competition for nutrients may have developed and led to a decreased concentration of *B. subtilis* in the co-culture or changed the metabolism of *B. subtilis*, leading to a reduction in the production of antibacterial factors.

When tested individually, the strains of *B*. *coagulans*, *B. pumilus* and *B. licheniformis* contained in the tested probiotic mix were not as effective as the *B. subtilis* strain in reducing *C. perfringens* concentrations. These findings do not imply that other strains of these *Bacillus* species may not be effective against *C. perfringens*, as some have been reported effective reducing the numbers of *C. perfringens* [21]. The lack of efficacy of the tested strains of *B. coagulans*, *B. licheniformis* and *B. pumilus*, individually or in combination, against *C. perfringens in vitro* suggests that they may not be ideal candidates for the control of *C. perfringens.*

Probiotic mixtures have been reported to be more effective than individual probiotics against gastrointestinal disorders in vitro and in vivo [22, 23]. However, meaningful comparisons between multi-strain and single-strain probiotics are scarce. Studies often compare multi-strain probiotics to single strains products that do not share the same bacterial species. In addition, comparisons are sometimes made with products containing different concentrations of bacteria, which generate results that can be difficult to interpret [24, 25]. A standardized method for comparing the efficacy of single versus multiple-strain probiotics is currently needed.

Compatibility between probiotic strains should be considered when designing multi-strain products for the control of *C. perfringens*. It is important to consider that different strains of probiotics may inhabit different segments of the intestinal tract and that the microbial interactions that limited the anticlostridial performance of *B. subtilis in vitro* may not occur in vivo. In addition, it is likely that interactions with the gut microbiome could also modify the behavior of these probiotics in vivo. Therefore, in vivo experimentation is necessary to confirm our observations.

In conclusion, the multi-strain probiotic product tested in this study did not decrease *C. perfringens* concentration in vitro using a co-culture method. Deconstruction of the probiotic blend showed that some of the individual probiotic strains used in this product were effective against *C. perfringens.* Among the tested species, *B. subtilis* was the most efficacious strain against *C. perfringens*. Combining other strains with *B. subtilis*, significantly decreased its anticlostridial efficacy.

### Limitations

The *Bacillus* strains reported in our experiment presented satisfactory growth under anaerobic conditions, however, the optimal growth of *Bacillus* is under aerobic conditions. This may affect interactions between bacterial species. Additionally, we tested the efficacy of the probiotics against *Clostridium perfringens* (ATCC 13,124 ™) to increase the reproducibility of the experiments. Therefore, different observations may be seen when testing different strains of *C. perfringens*, as well as different strains of *Bacillus*.

## Data Availability

Raw data and statistical analyses can be requested from Vinicius Buiatte (vub172@psu.edu) and Alberto Gino Lorenzoni (agl20@psu.edu). Experimental protocols can be requested from Vinicius Buiatte.

## List of abbreviations

ATCC: American Type Culture Collection
BS: Bacillus subtilis
BC: Bacillus coagulans
BL: Bacillus licheniformis
BP: Bacillus pumilus
